# The Whitefly *Bemisia tabaci* *Knottin-1* Gene Is Implicated in Regulating the Quantity of Tomato Yellow Leaf Curl Virus Ingested and Transmitted by the Insect

**DOI:** 10.3390/v8070205

**Published:** 2016-07-22

**Authors:** Aliza Hariton Shalev, Iris Sobol, Murad Ghanim, Shu-Sheng Liu, Henryk Czosnek

**Affiliations:** 1 Institute of Plant Sciences and Genetics in Agriculture, Robert H. Smith Faculty of Agriculture, Food and Environment, The Hebrew University of Jerusalem, Rehovot 76100, Israel; aliza.hariton@mail.huji.ac.il (A.H.S.); iris.sobol@mail.huji.ac.il (I.S.); 2 Institute of Plant Protection, Department of Entomology, Agricultural Research Organization, Volcani Center, Bet Dagan 50250, Israel; ghanim@volcani.agri.gov.il; 3 Institute of Insect Sciences, College of Agriculture and Biotechnology, Zhejiang University, Hangzhou, China; shshliu@zju.edu.cn

**Keywords:** gene silencing, insect-plant-virus interaction, geminiviruses, knottin genes

## Abstract

The whitefly *Bemisia tabaci* is a major pest to agricultural crops. It transmits begomoviruses, such as *Tomato yellow leaf curl virus* (TYLCV), in a circular, persistent fashion. Transcriptome analyses revealed that *B. tabaci* knottin genes were responsive to various stresses. Upon ingestion of tomato begomoviruses, two of the four knottin genes were upregulated, *knot-1* (with the highest expression) and *knot-3*. In this study, we examined the involvement of *B. tabaci* knottin genes in relation to TYLCV circulative transmission. Knottins were silenced by feeding whiteflies with knottin dsRNA *via* detached tomato leaves. Large amounts of *knot-1* transcripts were present in the abdomen of whiteflies, an obligatory transit site of begomoviruses in their circulative transmission pathway; *knot-1* silencing significantly depleted the abdomen from *knot-1* transcripts. *Knot-1* silencing led to an increase in the amounts of TYLCV ingested by the insects and transmitted to tomato test plants by several orders of magnitude. This effect was not observed following *knot-3* silencing. Hence, *knot-1* plays a role in restricting the quantity of virions an insect may acquire and transmit. We suggest that *knot-1* protects *B. tabaci* against deleterious effects caused by TYLCV by limiting the amount of virus associated with the whitefly vector.

## 1. Introduction

The whitefly *Bemisia tabaci* is a major agricultural pest worldwide, damaging plants by sucking their phloem and by transmitting many viruses [1]. Among them, the begomoviruses (genus *Begomovirus*, family *Geminiviridae*) are the most harmful. Begomoviruses are characterized by a 22 × 38 nm geminate particle. Their genome consists of one (monopartite) or two (bipartite) circular ssDNA components of approximately 2800 nucleotides each. *Tomato yellow leaf curl virus* (TYLCV) is one of the most destructive monopartite begomoviruses [2]. The TYLCV complex includes several species and numerous isolates [3]. The virion-sense strand comprises two genes, while the complementary-sense strand comprises four genes. The role of the encoded proteins was summarized elsewhere [4]. The TYLCV disease is managed by frequent applications of insecticides to contain the whitefly populations in the field and greenhouses, and by breeding resistant cultivars [2]. 

*B. tabaci* transmits begomoviruses in a circular, persistent fashion. Once ingested during feeding on the phloem of infected plant, virions pass along the food canal in the stylet and reach the esophagus and the gut. Virions can cross to the haemolymph via the filter chamber and the midgut [5]. From there virions reach the salivary glands [6] and are transmitted to plants with the saliva. In many reported cases, but not all (e.g., [7]), begomoviruses are deleterious to their whitefly host, reducing their longevity and fertility [8,9,10]. Examination of the transcriptome of *B. tabaci* provided an insight into the genes responsive to various stresses [11,12,13]. Knottins were among the genes identified upon ingestion of TYLCV [11,14] and bacteria [15], and during parasitization by the wasp *Eretmocerus mundus* [12]. Knottins are 59 to 65 amino acids proteins named as such because the three disulphide bonds in their sequences form a three-dimensional knot-like structure [16]. Knottins have been described in arthropods, mollusks, plants and mammals, where they function as antimicrobial peptides, toxins, or insecticides [17]. 

During the last decade, post-transcriptional gene silencing (PTGS) or RNA interference (RNAi) has proven to be an exquisite tool for deciphering gene function in insects [18,19,20]. The factors that determine RNAi stability and efficacy have been examined [21,22,23]. In the first study, involving *B. tabaci*, we demonstrated that by injecting into the body cavity dsRNA directed against genes uniquely expressed in the midgut and salivary glands, the amounts of targeted mRNA in the different organs were depleted by up to 70% [24]. To overcome the complications inherent to microinjecting such as traumatism and high mortality [24], we have developed a high throughput procedure to silence whitefly genes using a leaf-mediated dsRNA feeding approach [25]. This method was applied to explore the roles of genes within the molting hormone ecdysone synthesis and signaling pathway. Gene silencing reduced survival and delayed development of the whitefly nymphal stages [25], demonstrating that disruption of whitefly gene expression may help control the deleterious effects of this insect.

In this work, we applied the leaf-mediated silencing approach to investigate the function of knottin genes in the interaction between TYLCV and its whitefly vector. Previous analyses of cDNA libraries of non-viruliferous whiteflies and of whiteflies that fed on tomato plants infected with the begomoviruses TYLCV and *Tomato mottle virus* revealed a family of four knottin-like genes, coined *knot-1* to *-4*. *Knot-1* and *knot-3* were more abundant in the cDNA libraries from viruliferous than from non-viruliferous whiteflies [11,14]. Therefore, we investigated the role of these genes in *B. tabaci* ingestion and transmission of TYLCV. We found that silencing *knot-1* increased by several orders of magnitude the quantity of virus acquired by the insects and transmitted to tomato test plants. Hence, it seems that *knottin-1* is involved in restraining the amount of TYLCV particles acquired by *B. tabaci* during feeding and during the circulation of the virus in the insect body.

## 2. Materials and Methods

### 2.1. Plants and Whiteflies

Tomato plants (*Solanum lycopersicum* cv. Daniella) were grown in a greenhouse under controlled conditions. Whiteflies (*Bemisia tabaci* Middle East-Asia Minor 1, previously known as “B biotype” [26], were maintained on tomato plants (cv. Daniella) in insect-proof wood-framed cages in a climate-controlled room (14 h/8 h light/dark, 24–27 °C). Whitefly adults, seven days after emergence, were used to inoculate TYLCV tomato plants (cv. Daniella) at the 5–6 true leaf stage.

### 2.2. Isolation of RNA from *B. tabaci*, Preparation of cDNA and Quantitative RT-PCR (qPCR)

Total RNA was extracted from whiteflies using the TRI-reagent method (Merck, Grand Island, NU, USA) and quantified with a NanoDrop spectrophotometer (Thermo Fischer Scientific, Wilmington, DE, USA). RNA quality was appraised by subjecting the RNA samples to gel electrophoresis to verify rRNAs integrity. RNA was reverse transcribed using the EZ-First Strand cDNA synthesis Kit (Biological Industries, Bet-Haemek, Israel) with oligo-dT primers in a total reaction volume of 20 µL. The amount of transcripts was appraised by qPCR in three technical replicates for each of three biologically independent experiments. qRT-PCR was performed using the Rotor-Gene 6000 machine (Corbett Robotics Pty Ltd., Brisbane, Australia) with SYBR-Green detection (SYBR^®^ Premix Ex Taq^TM^II, Takara, Kyoto, Japan). The accompanying software (Rotor Gene Q-Series Software V2.0) was used for qPCR data normalization and quantification. The whitefly β*-actin* gene was used as a calibrator gene (see primers used below). Similar levels of β-*actin* transcripts were obtained in both the dsRNA-treated and control whiteflies (data not shown), indicating that the expression of β*-actin* was not affected by the dsRNA treatment. The expression of each gene was tested in two technical replicates for each of three biologically independent experiments. The ∆∆Ct method for was applied for relative quantifications. All the primers generated a single peak in the real-time dissociation analysis and had the amplification efficiencies ranging from 0.95 to 1.0. 

### 2.3. Isolation of DNA from *B. tabaci* and Tomato Plants; Measurement of TYLCV Amounts

Adult whiteflies were collected using an aspirator and stored at −80 °C until DNA extraction. DNA from groups of 25 insects was extracted using CTAB [27], including RNase treatment. Young leaves were collected from plants and DNA was extracted using the Wizard^®^ genomic DNA purification Kit (Promega, Madison, WI, USA) following the manufacturer’s instructions. DNA was dissolved in 100 µL Rehydration buffer and its quality was appraised following agarose gel electrophoresis. The DNA concentrations were measured using a NanoDrop spectrophotometer. The amounts of TYLCV were measured by qPCR using TYLCV CP-specific primers in the presence of SYBR Green I (Takara, Kyoto, Japan) in a Corbett Research Rotor-Gene 6000 cycler. The reaction was as follows: 30 s at 94 °C followed by 40 cycles consisting of 10 s at 94 °C, 30 s at 59 °C, and 20 s at 72 °C. The primers used to amplify a 200 bp fragment of TYLCV were sense: 5′-GAAGCGACCAGGCGATATAA-3′ and anti-sense: 5′-GGAACATCAGG GCTTCGATA-3′. A 60 bp fragment of the tomato β*-actin* gene served as an internal reference; it was amplified using the primer pair: sense: 5′-TGGAGGA TCCATCCTTGCATCAC-3′ and complementary sense: 5′-TCGCCCTTTGAAAT CCACATCTGC-3′. A 200 bp fragment of the whitefly β*-actin* gene also served as an internal reference; it was amplified using the following primer pair: sense: 5′-TCTTCCAGCCATCCTTCTTG-3′ and complementary sense: 5′-CGGTGATTTC CTTCTGCATT-3′. For absolute quantification, standard curves were generated using ten-fold dilutions of a CP-carrying plasmid of known size and concentration.

### 2.4. dsRNA Synthesis

**Figure 1:**
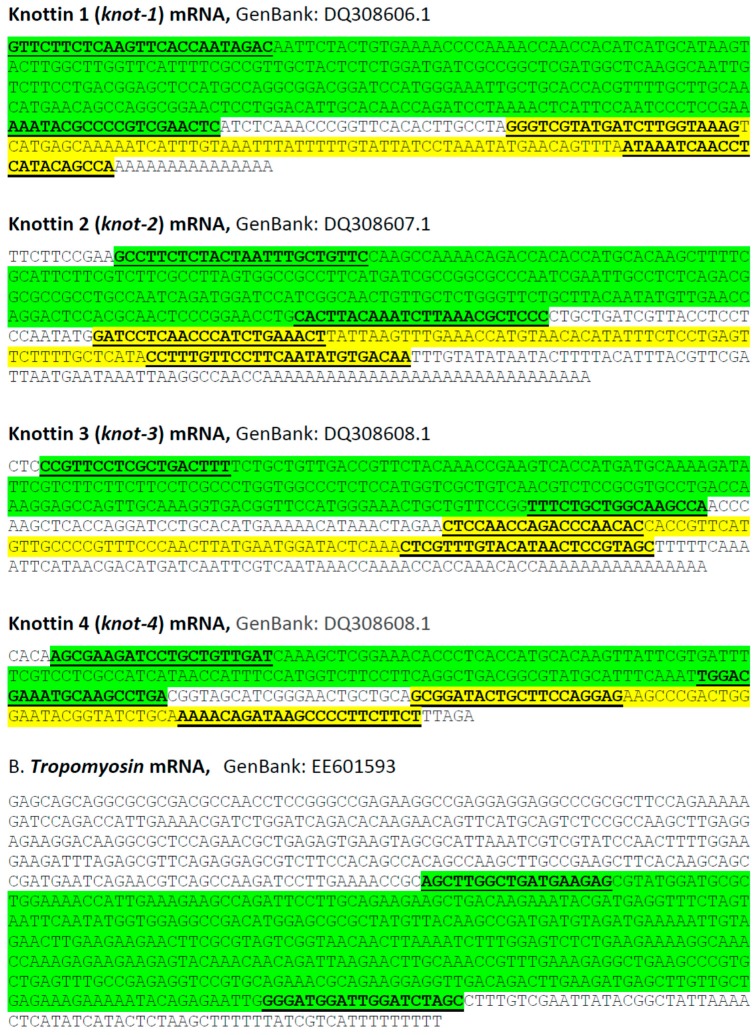
Sequences of whitefly knottin genes. Sequences retrieved from GenBank. The whitefly tropomyosin gene served as control for gene silencing. The primers used to amplify the gene fragment used for dsRNA synthesis (green) and for measuring expressing by qPCR (yellow) are underlined.

Primers for the synthesis of double stranded RNA were designed based on the sequences of the relevant genes (Figure 1). dsRNAs were synthesized using the AmpliScribe™ T7-Flash™ Transcription Kit (Epicentre Biotechnologies, Madison, WI, USA) as described previously [24]. The dsRNA obtained was diluted with nuclease-free water to a final concentration of 0.5 µg/µL. The dsRNA purity and quality was determined by agarose gel electrophoresis. 

To follow its path in leaf and insect, the dsRNA was Cy-labeled as follows. The candidate gene was subjected to PCR using specific primers conjugated to 27 bases of the T7 RNA polymerase promoter. PCR products were then purified (QIAquick PCR Purification Kit, Qiagen, Hilden, Germany) and used as template for dsRNA in vitro synthesis using the Ampliscribe T7 RNA transcription kit as above, and a fluorescent ribonucleotide (dCTP-cy3 at 2 mM with unlabeled 8 mM dCTP) for generating fluorescently labeled dsRNA molecules. The dsRNA was purified (RNeasy Mini Kit, Qiagen) and quantified using a spectrophotometer. Leaflets were soaked in tubes containing 0.5 mL of dsRNA at the concentration of 1 µg/µL labeled dsRNA in sterile water. Ingested dsRNA reached the leaf vascular system and was detected in the digestive system of insects feeding on these leaflets (Supplemental Figure S1, panels 3 and 5). 

### 2.5. Gene Silencing by Leaf-Mediated dsRNA Feeding

A tomato leaflet was cut off from a tomato plant and placed in a 1.5 mL Eppendorf tube containing 0.5 mL of dsRNA at the concentration of 0.5 µg/µL for each targeted gene (distilled water was used as control). Then the tubes containing the dsRNA and leaves were transferred into a 50 mL plastic tube (Corning Inc., Corning, NY, USA) (Supplementary Figure S1, panel 2). Adult whiteflies were released into this silencing system and the tube was covered with a piece of paper towel tightly held with a rubber band [25]. The whiteflies were left to feed on the leaflet for two days. Three replicates were established in each of the treatments and the controls. After two days of feeding, samples (each containing 25 adult whiteflies) were collected for gene expression and silencing analysis by qRT-PCR as described above. The remaining whiteflies were transferred to a TYLCV infected leaf for two more days and then the whitefly samples were again taken for analysis to determine gene expression and silencing and amounts of TYLCV acquired. Three whiteflies were put in a cage and clipped to a leaf of a young tomato test plant (5–6 true leaf stage). Whiteflies were discarded after one day and the leaf was removed after one week.

### 2.6. Fluorescence in Situ Hybridization (FISH)

FISH was performed as previously described [28]. Briefly, specimens were fixed overnight in Carnoy’s fixative (chloroform-ethanol-glacial acetic acid, 6:3:1 (vol/vol/vol), decolorized in 6% H_2_O_2_ in ethanol for 2 h, washed in 100% ethanol, and hybridized overnight in hybridization buffer (20 mM Tris-HCl (pH 8.0), 0.9 M NaCl, 0.01% SDS, 30% formamide) containing 10 pmol of fluorescent probes per mL. The primer used for specific targeting of *knottin-1* transcripts was Cy3-knot-1 (5′-Cy3-GTTCTTCTCAAGTTCACCAATAGAC-3′). The stained samples were mounted whole in hybridization buffer and viewed under an IX81 Olympus FluoView500 confocal microscope. The specificity of detection was appraised by performing the procedure without the probe. 

### 2.7. Statistical Analysis of Data

For all statistical analyses, JMP software (Release 5.0, SAS Institute, Inc.). All data were subjected to one-way ANOVA. The significant differences among means were evaluated by application of the Tukey-Kramer honestly significant difference (HSD) test at *p* < 0.05. The data are presented as means ± SEM. In all of the figures, an asterisk was used to indicate significance between treatments.

## 3. Results

### 3.1. Out of the Four Whitefly Knottin Genes, *knot-1* Shows the Highest Levels of Expression. It Is Upregulated upon Ingestion of TYLCV

The sequences of the *B. tabaci* knottin genes (*knot-1* to -*4*) were retrieved from GenBank (accession numbers DQ308606.1, DQ308607.1, DQ308608.1, DQ308609.1, respectively) (Figure 1). Two different sets of primers were designed: one for the synthesis of silencing dsRNA, and the other for monitoring the amounts of *knot* transcripts by RT-qPCR (qPCR). For each gene, the region used for qPCR was separated (downstream) from the region that served as template for dsRNA synthesis.

**Figure 2:**
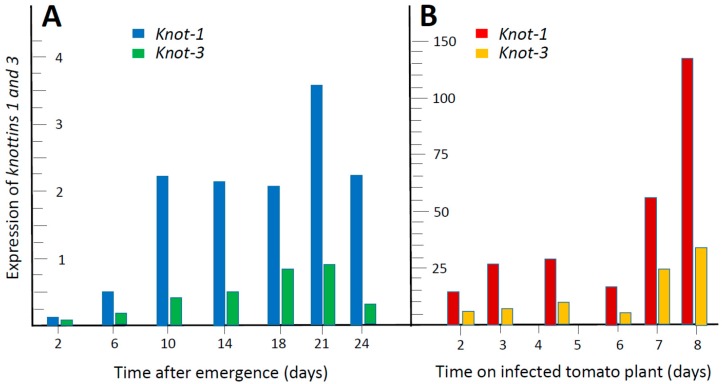
Expression of *knot-1* and *knot-3* during adult whitefly development and upregulation by *Tomato yellow leaf curl virus* (TYLCV). (**A**) qPCR quantification of transcripts (relative to β-*actin*) in synchronized whitefly populations reared on tomato plants, collected at the time indicated after emergence; (**B**) Five- to seven-day-old whiteflies were caged with leaves of TYLCV-infected tomatoes and the amounts of transcripts were estimated by qPCR (relative to β-*actin*) at the time indicated thereafter. Pools of about 50 insects were used for each time point.

The amounts of *knottin* transcripts (relative to β*-actin*) were followed by qPCR during the adult life of the insect (2, 6, 10, 14, 18, 21 and 24 days after emergence), using synchronized populations. Pools of 50 insects were used for each time point. Figure 2A shows that the expression of *knot-1* and *knot-3* increased upon aging, especially during the first 10–14 days after emergence. At all times, the levels of *knot-1* transcripts were two to four times higher than those of *knot-3.* The levels of *knot-4* were about one twentieth those of *knot-1*; they were too low to clearly point to a developmental pattern. *Knot-2* transcripts were undetectable. 

The effect of TYLCV on the expression of *knot-1* and *knot-3* was appraised (Figure 2B). Five to 7 days-old adult whiteflies were caged with leaves of TYLCV-infected tomatoes and the amounts of *knot* transcripts were estimated by qPCR 2, 3, 4–5, 6, 7 and 8 days thereafter. The presence of the virus was associated with a large increase (20-fold and above) in the expression of *knot-1* and knot-3 (*knot-1* remaining higher than *knot-3*).

### 3.2. Feeding Whiteflies on Tomato Leaflets Bathing in dsRNA Targeted against *knot-1* and *knot-3* Downregulated These Genes by about Two Thirds

The whitefly *knottin* genes were silenced by releasing whiteflies (5–7 days after emergence) into the silencing system consisting of detached tomato leaflets soaking in dsRNA directed against specific *knottin* transcripts (or in water as control). The flow chart of the experiments described in this article is shown in Supplemental Figure S1. We tested the effect of dsRNA at concentrations of 0.1, 0.5 and 1 μg/μL and found that 0.5 μg dsRNA/μL provided an optimal silencing effect in this system. We have shown previously that the dsRNA was readily detected in the tomato leaflets one day after the beginning of the treatment. Moreover, the dsRNA was stable in the aqueous solution and in the tomato leaflet for at least five days [25]. Feeding whiteflies on leaflets soaking in dsRNA (directed against *knottins* or any other gene, e.g., *Tropomyosin*, used here as an unrelated control) or water, did not significantly affected the survival of the insects.

**Figure 3:**
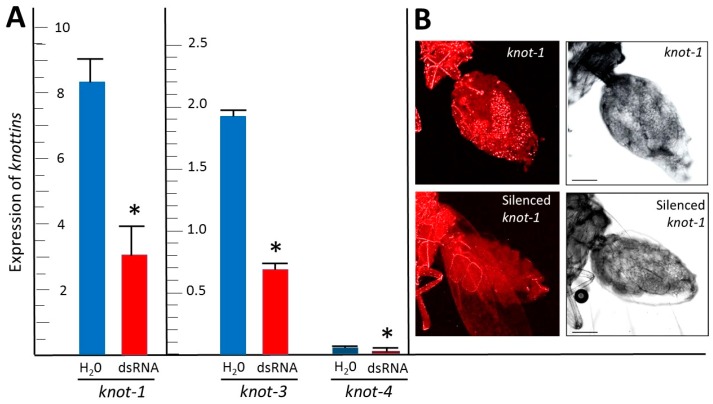
Expression of members of the whitefly Knottin gene family in insects: effect of gene silencing. (**A**) qPCR quantification of *knot-1*; *-3* and *-4* transcripts (relative to β-*actin*) in adult whiteflies (collected 7 days after emergence) after caging the insects for 48 h with tomato leaflets bathing in water (H_2_O) or in *knot-1*; *-3* and *-4* dsRNA (dsRNA); (*n* = 3; mean ± SEM). An asterisk above the bars indicates significant differences between treatments at *p* < 0.05. (**B**) Confocal microscopy (FISH) of *knot-1* transcripts in the abdomen of female whiteflies; before (upper panels) and after (lower panels) *knot-1* silencing; using a Cy3-labeled *knot-1* primer. Left picture: bright field; right picture: red signal associated with the presence of *knot-1* transcripts. Bar: 100 μm.

After the whiteflies had fed on tomato leaflets bathing in *knot-1* dsRNA, the amount of *knot-1* transcript in their body was about one third that of whiteflies feeding on leaves soaking in water (Figure 3A). Similarly, feeding whiteflies on leaflets soaking in *knot-3* and *knot-4* depleted the amounts of *knot-3* and *knot-4* transcripts by approximately two-thirds. These results were confirmed by visualizing *knot-1* transcripts in the abdomen of female whiteflies by FISH (Figure 3B). The *knot-1* transcript signal conspicuous in the abdomen of female whiteflies was greatly reduced upon *knot-1* silencing.

### 3.3. Silencing *knot-1* Has no Effect on the Expression of the Other Whitefly Knottin Genes

**Figure 4:**
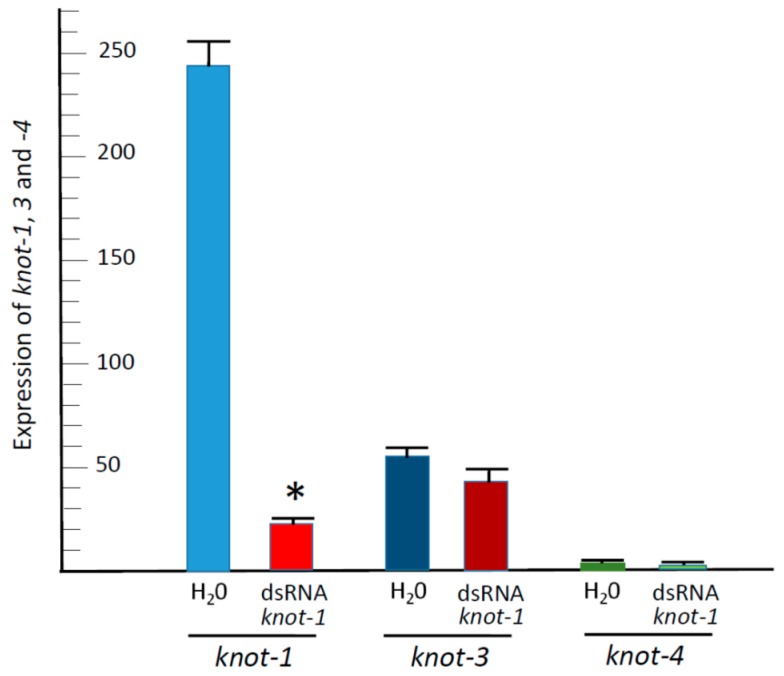
Expression of *knot-1; -3;* and *-4* upon *knot-1* silencing. qPCR quantification of *knot* transcripts (relative to β*-actin* transcripts) from whiteflies caged for 48 h with tomato leaflets bathing in water (H_2_O) or in *knot-1; -3* and -*4* dsRNA (dsRNA); (*n* = 3; mean ± SEM). An asterisk above the bars indicates significant differences between treatments at *p* < 0.05.

Since the *knottin* genes are members of the same family, we have measured the effect of *knot-1* silencing on the expression of *knot-1*, *knot-3* and *knot-4* (Figure 4). While upon *knot-1* silencing the expression of *knot-1* was reduced by approximately 90%, *knot-1* silencing did not significantly down-regulate the expression of *knot-3* and *knot-4*. Hence, it is likely that the expression of the four knottin genes is not interconnected in a way where perturbations in one does not influence each other significantly. 

### 3.4. Whiteflies with Silencing *knot-1* Contain Increased Amounts of TYLCV upon Feeding on Leaves of Infected Tomato Plants

**Figure 5:**
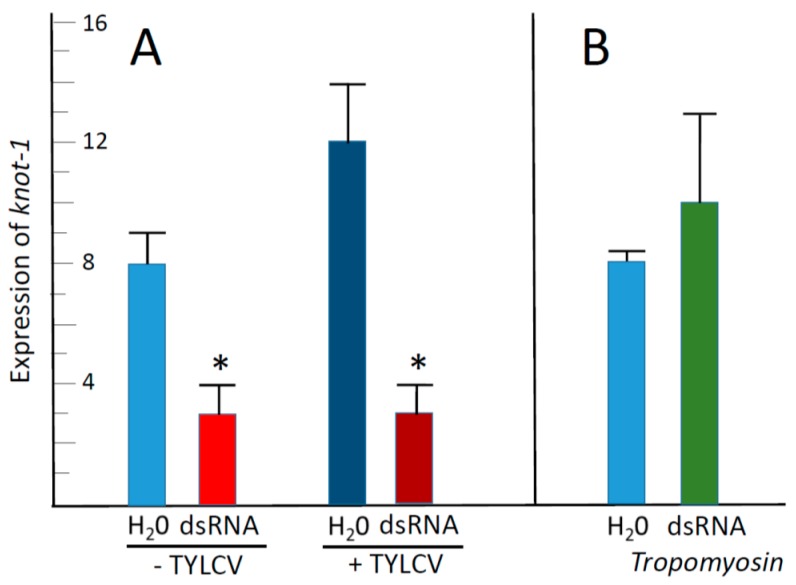
Effect of TYLCV acquisition on the expression of *knot-1*; in whiteflies with and without silenced *knot-1*. (**A**) qPCR quantification of *knot-1* transcript levels in adult whiteflies after caging the insects for 48 h with tomato bathing in water (H_2_O) or in *knot-1* dsRNA (dsRNA); before (− TYLCV) and after caging with infected tomato leaflets (+ TYLCV) for a 48 h virus acquisition (*n* = 3; mean ± SEM). (**B**) *knot-1* expression in whiteflies following caging the insects for 48 h with tomato bathing in water (H_2_O) or in *tropomyosin* dsRNA (dsRNA *tropomyosin*) (*n* = 3; mean ± SEM). An asterisk above the bars indicates significant differences between treatments at *p* < 0.05.

Following feeding on leaflets soaking in water and on *knot-1* dsRNA as described above, the insects were caged with TYLCV-infected leaflets for a virus acquisition access period of 48 h, after which the expression of *knot-1* was estimated. In the unsilenced whiteflies, the presence of TYLCV was accompanied by an increase of about one third in the level of *knot-1* expression (Figure 5A). In comparison, the presence of TYLCV in *knot-1*-silenced whiteflies did not change the expression levels of *knot-1*. Altogether, in viruliferous insects, the expression levels of *knot-1* in silenced whiteflies were about one third those measured in unsilenced whiteflies (Figure 5A). 

To eliminate the possibility that dsRNA by itself (directed against any gene) affected the expression of *knot-1*, whiteflies were caged for two days with tomato leaflets bathing in water and in dsRNA directed against the whitefly *Tropomyosin* gene. Then the levels of *knot-1* expression were measured. Figure 5B shows that whitefly treatment with tropomyosin dsRNA did not significantly affect the expression of *knot-1*, indicating that the downregulation of *knot-1* was specifically the result of targeting *knot-1* transcripts with *knot-1* dsRNA. 

**Figure 6:**
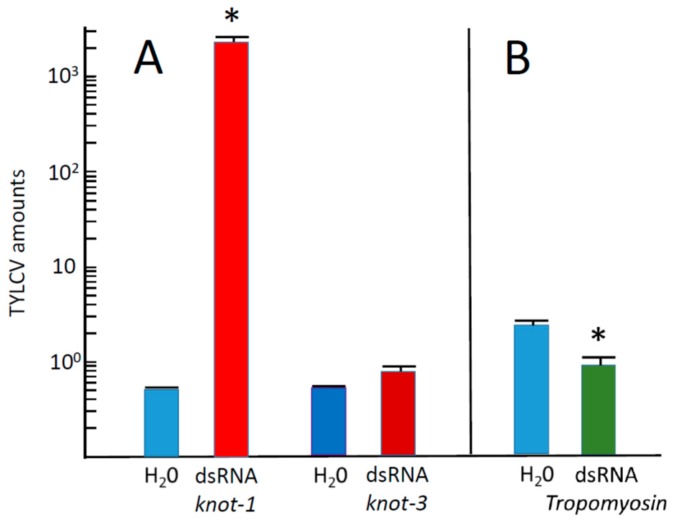
TYLCV amounts acquired by whiteflies with down-regulated *knot-1* and *knot-3* during a 48 h acquisition period on infected tomato leaves. (**A**) Viral amounts (viral genomes per insect β*-actin* genes) in whiteflies caged with down-regulated *knot-1* (dsRNA *knot-1*) or *knot-3* (dsRNA *knot-3*) compared with control not-silenced insects (H_2_O); (**B**) viral amounts in whiteflies caged with down-regulated *tropomyosin* (dsRNA *tropomyosin*) compared with control insects (H_2_O) (*n* = 3; mean ± SEM). An asterisk above the bars indicates significant differences between treatments at *p* < 0.05.

The effect of *knot-1* silencing on the amount of TYLCV acquired by whiteflies during the 48 h feeding period was appraised (Figure 6). Whiteflies with silenced *knot-1* acquired three orders of magnitudes more virus than untreated insects (Figure 6A). By comparison, whiteflies with silenced *knot-3* acquired about the same amount of virus than untreated whiteflies. Insects with silenced *Tropomyosin* acquired 2–3 times less virus than untreated insects (Figure 6B). It is possible that muscle proteins such as tropomyosin, myosin and actin are also involved in TYLCV acquisition and movement in the insect body, an observation worth further investigation. These results indicated that the effect of *knottin* silencing on the acquisition of TYLCV were *knot-1*-specific. 

### 3.5. Tomato Plants Inoculated by Viruliferous Whiteflies with Silenced *knot-1* Presented Early Disease Symptoms and Contained Large Amounts of Virus

**Figure 7:**
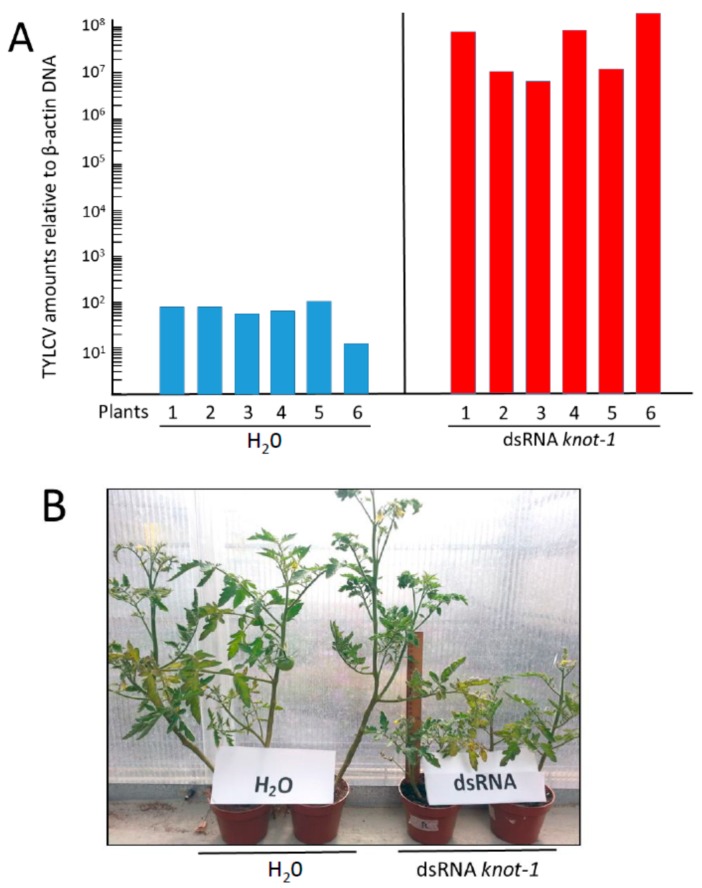
TYLCV amounts and symptoms in tomato plants infected by viruliferous whiteflies without (H_2_O) and with *knot-1* silenced gene. (**A**) Viral amounts (viral genomes per plant β*-actin* gene) in tomato plants 19 days after infection by viruliferous whiteflies without (H_2_O) and with *knot-1* silenced (dsRNA *knot-1*); (**B**) symptom appearance of tomato plants 24 days after infection with not-silenced (H_2_O) and *knot-1* silenced insects (dsRNA *knot-1*).

Following TYLCV acquisition, the two groups of insects (*knot-1* silenced and not silenced) were caged with young leaves of tomato test plants (at the 5–6 true leaf stage) for a 24 h inoculation access period, 3 insects in a clip cage per plant (minimal conditions to ensure nearly 100% infection [29]. The amount of viral DNA associated with the test plants was measured 5, 9, 13, 16 and 19 days post-inoculation. Nineteen days after whitefly-mediated inoculation, the plants infected by the silenced *knot-1* insects contained five orders of magnitude more virus than the control plants (Figure 7A). The typical disease symptoms of arrest of growth (61 vs. 90 cm high in average), yellowing and curling of upper leaves (Figure 7B) started to appear about 7–10 days earlier in plants infected by *knot-1* silenced insects than by control whiteflies (12 vs. 21 days). These results indicated that the large amounts of TYLCV acquired by the *knot-1* silenced whiteflies do not stay confined in the insect body, disconnected from virus the circulative pathway, but are transmitted to plants during feeding.

### 3.6. Inoculation of Tomato Plants by Whiteflies with Silenced *knot-3* Significantly Modified Neither the Time of Symptom Appearance nor the Accumulation of TYLCV

**Figure 8:**
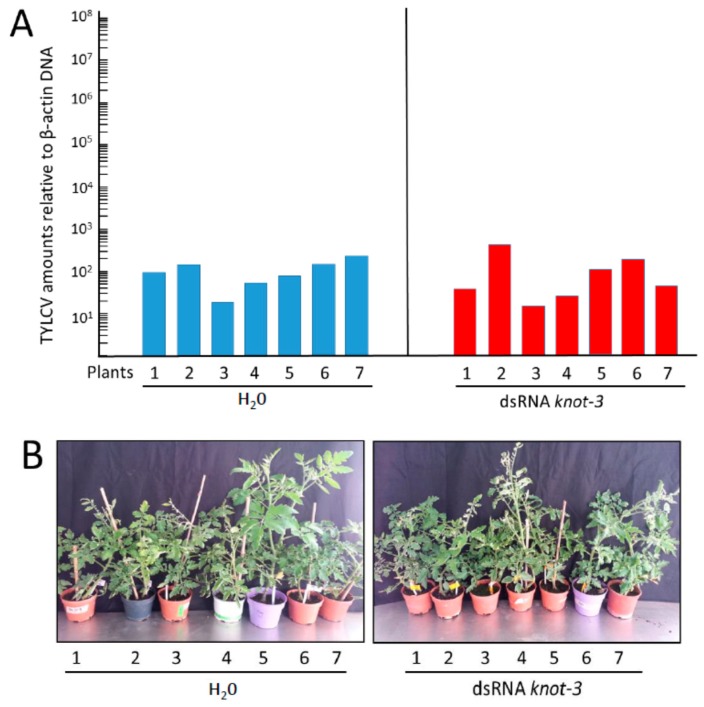
TYLCV amounts and symptoms in tomato plants infected by viruliferous whiteflies without (H_2_O) and with silenced *knot-3.* (**A**) TYLCV amounts (viral genomes per plant β*-actin* genes) in tomato plants 19 days after infection by viruliferous whiteflies without (H_2_O) and with silenced *knot-3* (dsRNA *knot-3*); (**B**) symptom appearance of tomato plants 24 days after infection with not-silenced (H_2_O) and *knot-3* silenced insects (dsRNA *knot-3*).

The specificity of *knot-1* in restraining virus acquisition and transmission was tested by comparing the effect of silencing *knot-1* and *knot-3*. Upon feeding on tomato leaflets soaking in *knot-3* dsRNA, *knot-3* expression was reduced by about 2/3 (Figure 3A). The *knot-3* silenced whiteflies contained approximately the same amount of virus than the non-treated insects (Figure 6A), in contrast to the three orders of magnitude observed between *knot-1* silenced and control insects (Figure 7A). The *knot-3* silenced whiteflies were able to transmit the TYLCV disease to tomato tests plants. However, contrary to the tomatoes infected with viruliferous *knot-1* silenced whiteflies, which contained five to six orders of magnitude more virus than the plants infected by the control insects (Figure 7A), the plants infected with viruliferous *knot-3* silenced whiteflies contained approximately the same amounts of viral DNA as the plants infected by viruliferous, untreated insects (Figure 8A). The plants presented similar symptoms of stunting and yellowing (Figure 8B). These results strongly supported the unique role of *knot-1* in controlling the amount of TYLCV in the whitefly body.

## 4. Discussion

We examined the involvement of members of the whitefly *knottin* gene family in the interaction of TYLCV with its *B. tabaci* vector, applying RNAi-based gene silencing. The flow chart in Supplemental Figure S1 summarizes the experiments. The factors for a successful use of RNAi in insect science such as targeted species, choice of gene, mode of delivery, dsRNA dosage, have been reviewed recently [30]. To circumvent potential effects due to insect aging and different plant allelochemicals, we used synchronized whitefly populations raised on the very same tomato genotypes that were utilized for leaf-mediated silencing. The procedure we used to silence *knottins* (Supplemental Figure S1, panels 2 and 4) was developed to silence genes involved in whitefly nymphal development [25]. The uptake of dsRNA by the leaf vascular system and by the insects feeding on these leaves was visualized using Cy-labeled dsRNA (Supplemental Figure S1, panels 4 and 5). Several methods of dsRNA delivery have been applied to silence insect genes: by microinjection [24,31], with the diet [32,33], by topical application [34], by feeding on leaves of dsRNA-expressing transgenic plants [35,36], and by leaflet absorption [37]. In the latter case, similarly to our own experiments, leaflets were immersed in 200 µL water containing 10 µg dsRNA of various target genes. 

Leaf-mediated feeding of *knottin*-specific dsRNA by whiteflies was associated with a downregulation of the *knottin* genes by more than 50%, whether *knot-1*, *-3* or *-4* (Figure 3A). The silencing of a given *knottin* gene was specific although the sequences of the *knottin* genes present a high level of similarity (Figure 1). Silencing of *knot-1* did not affect the expression of *knot-3* and *knot-4* (Figure 4). Moreover, the silencing of *knot-1* was specific and did not occur when an unrelated gene, such as *tropomyosin*, was silenced (Figure 5B). 

Ingestion of virus led to the upregulation of *knot-1* (and to a much lesser extent of *knot-3*) suggesting that this gene is responsive to the stress caused by the virus. In this perspective, silencing of *knot-1* neutralized the stress-response to TYLCV (Figure 2 and Figure 5). As a result, whiteflies with depleted levels of *knot-1* contained several orders of magnitude more virus than non-silenced insects (Figure 6). This phenomenon was specific for the *knot-1* gene, and did not occur when *knot-3* (or *tropomyosin*) was silenced. Tomato plants inoculated by *knot-1* silenced insects contained several orders of magnitude more virus than plants inoculated with non-silenced insects. Moreover, the disease symptoms, stunting, leaf yellowing and curling, appeared 7 to 10 days earlier and were more pronounced (Figure 7). Silencing *knot-3* was not accompanied by the severe effects associated with *knot-1* silencing, such as a dramatic increase of virus acquisition and transmission to tomato plants (Figure 8). Although the amounts of virus inoculated by the different types of viruliferous whiteflies during the 24 h access feeding period are unknown, the results presented in Figure 7 and Figure 8 showed a direct correlation between the amounts of virus in the whiteflies and the amounts of virus in the plants inoculated by these whiteflies. These results suggest that the additional amounts of virus in *knot-1* silenced whiteflies are not removed from the circulative pathway but are transmitted in a manner similar to the virus transmitted by unsilenced insects.

Since *knot-1* transcripts are found mainly in the whitefly abdomen (Figure 3B), it is tempting to suggest that *knot-1* regulates the number of virions in the hemolymph, an obligatory site of begomovirus transit from the digestive tract to the salivary glands [5]. How this control is exerted is not known. Knottins are present in diverse organisms and possess various biological functions. For instance, knottins from pea (*Pisum sativum*) have insecticide properties to certain species of cereal weevils, mosquitoes and aphids [38]; the venom of the spider *Segesteria florentina* contains the insecticidal toxin Sf1a, a knottin that blocks the pore of insect voltage-gated sodium channels [39]. Knottins from the beetle *Psacothea hilaris* present antimicrobial properties towards Gram-positive and Gram-negative bacterial strains [40]. *Knot-1* may be involved in maintaining the amount of virions associated with the whitefly to a level where it is not overwhelmingly harmful to the insect. We are not aware of any other case of knottin or knottin-like genes directly or indirectly involved in the regulation of virus amounts in animal or plant cells. 

We have previously suggested that some begomoviruses interact actively with its *B. tabaci* vector, to a point reminiscent of a host-pathogen relationship [41,42]. Indeed, in many instances (but not always) the long-term presence of begomoviruses (sometimes for the entire life) has deleterious effects on the longevity and fertility of the whitefly host [9]. In addition, begomoviruses affect the insect pattern of gene expression [43], and may replicate to some extent in their host [44]. It is possible that the geminivirus-vector partnership evolved towards a neutralization of any viral function that may negatively affect the insect. In this perspective, the role of the insect knottins, in particular *knot-1*, may be to regulate the amount of virus uptaken by the insect to levels the insect can handle without causing extensive deleterious effects. The long-term effect of *knot-1* silencing on the biology of viruliferous whitefly, especially on longevity and fertility, warrants further research. 

Beside *knot-1*, we expect that other genes are involved in the control of begomovirus circulative transmission. The comparison of the transcriptome of viruliferous vs. non-viruliferous whiteflies has identified a large number of genes upregulated and dowregulated upon TYLCV acquisition organized in 157 differentially regulated pathways [13,43]. In addition, TYLCV was associated with the activation of the whitefly immune responses, including autophagy (Atg3, 9 and 12), lysosome proteins (AP-1, cathepsin B and D, protein tyrosine phosphatase, saposin, phosphatidylcholine acyltransferase), melanization (dopa carboxylase) and antimicrobial peptides (including knottin-3). Hsp70 was strongly upregulated upon acquisition of TYLCV and of the bipartite *Squash leaf curl begomovirus* [28]. The wealth of publication on the use of RNAi to downregulate insect genes indicated that a strategy based on targeting whitefly genes involved in begomovirus transmission could be used to control the virus before it could be transmitted and infect target crops. 

## Supplementary Materials

The following figure is available online at http://www.mdpi.com/1999-4915/8/7/205/s1. **Figure S1.** Flow of described experiments.
