# Supplementary material for: The Whitefly Bemisia tabaci Knottin-1 Gene Is Implicated in Regulating the Quantity of Tomato Yellow Leaf Curl Virus Ingested and Transmitted by the Insect
Source: Viruses. 2016 Jul 22;8(7):205. doi: 10.3390/v8070205 (PMC4974540; doi:10.3390/v8070205)
Supplement: Supplementary file 1 [file viruses-08-00205-s001.pdf]

Supplementary Materials: The Whitefly *Bemisia tabaci Knottin-1* Gene Is Implicated in Regulating the Quantity of Tomato Yellow Leaf Curl Virus Ingested and Transmitted by the Insect

Aliza Hariton Shalev, Iris Sobol, Murad Ghanim, Shu-Sheng Liu and Henryk Czosnek

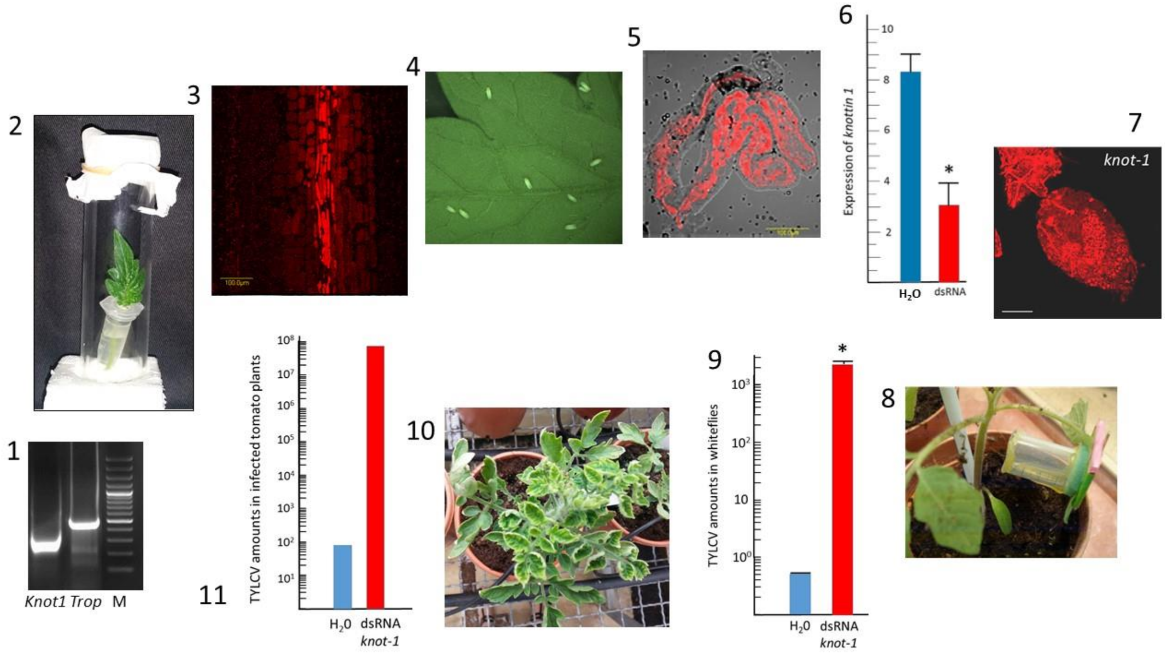

Figure S1. Flow of described experiments. (**1**) Agarose gel electrophoresis of dsRNA synthesized using the knottin 1 and tropomyosin specific primers shown in Figure 1; (**2**) Tomato leaflet placed in a 1.5 mL Eppendorf tube containing dsRNA placed in a 50 mL plastic tube; adult whiteflies were released into this silencing system and left to feed on the leaflet for two days; (**3**) Translocation of Cy-3 labeled dsRNA in the leaflet vascular system—confocal image of longitudinal section of petiole; (**4**) Whiteflies feeding on the tomato leaflet; (**5**) FISH of Cy-3 labeled dsRNA in the dissected gut of a whitefly that fed on a tomato leaflet bathing in Cy-3 labeled dsRNA; (**6**) Depletion of knottin 1 transcripts in whiteflies that fed on tomato leaflets bathing in knottin 1 dsRNA; and (**7**) confocal visualization of knottin 1 transcripts in the abdomen of the treated whiteflies; (**8**) Whiteflies caged with leaf of TYLCV-infected tomato plant for a 2-days virus acquisition period; and (**9**) viral DNA amounts ingested; (**10**) Infection of tomato test plants with treated viruliferous whiteflies and development of viral disease symptoms; (**11**) qPCR measurement of virus amounts in infected tomato plants.
